# Optimizing endectocide and ectocide compound evaluation in *Anopheles* malaria vectors

**DOI:** 10.1186/s13071-025-07040-2

**Published:** 2025-10-17

**Authors:** Kevin C. Kobylinski, Thitipong Hongsuwong, Pattarapon Khemrattrakool, Natpapat Kaewkhao, Rattawan Kullasakboonsri, Theerawit Phanphoowong, Patchara Sriwichai, Borimas Hanboonkunupakarn, Podjanee Jittamala, Joel Tarning

**Affiliations:** 1 Mahidol Oxford Tropical Medicine Research Unit, Faculty of Tropical Medicine, Mahidol University, Bangkok, Thailand; 2 Department of Medical Entomology, Faculty of Tropical Medicine, Mahidol University, Bangkok, Thailand; 3 Department of Clinical Tropical Medicine, Faculty of Tropical Medicine, Mahidol University, Bangkok, Thailand; 4 Department of Tropical Hygiene, Faculty of Tropical Medicine, Mahidol University, Bangkok, Thailand; 5 Centre for Tropical Medicine and Global Health, Nuffield Department of Clinical Medicine, University of Oxford, Oxford, UK

**Keywords:** *Anopheles*, Blood, Blood meal, Dilution, Endectocide, Ivermectin, Midgut, Plasma, Sugar, Survival

## Abstract

**Background:**

Mass endectocide or ectocide treatment of humans or livestock has been suggested as a possible malaria vector control tool. This work provides guidance for in vitro endectocide and ectocide experiments and raises biological points for further evaluation.

**Methods:**

Three experiments with ivermectin were performed with *Anopheles dirus* and *Anopheles minimus*. The first experiment assessed the impact of a sugar diet (10% sucrose, “*Sucrose*”; multivitamin syrup, “*Multivitamin*”; or multivitamin syrup followed by 10% sucrose, “*Mix*”) on mosquito mortality following ingestion of a range of ivermectin-spiked blood meal concentrations. The lethal concentrations that kill 50% (LC_50_) and 90% (LC_90_) of mosquitoes were estimated using a normalized concentration–response analysis (IC_50_ and Hill slope). The second experiment assessed the impact on mosquito mortality after ingesting ivermectin spiked into a plasma meal or a blood meal that was either fresh or previously frozen. Log-rank survival curve analysis (Mantel–Cox method) was used to compare mosquito survival between groups. The third experiment sought to quantify the concentration of ivermectin in a blood meal compared to the amount imbibed into the mosquito midgut as measured by liquid chromatography–tandem mass spectrometry (LC–MS/MS).

**Results:**

The Multivitamin diet was found to substantially increase *An. dirus* LC_50_ compared to Sucrose and Mix diets, while the Sucrose diet had reduced control survival post-blood meal for both *An. dirus* and *An. minimus*. Ivermectin mortality response was substantially increased when ingested in a blood meal compared to a plasma meal for *An. dirus*, while the inverse was observed for *An. minimus*. For both *An. dirus* and *An. minimus*, an approximately 20% loss in ivermectin concentration was observed in the midgut compared to the blood meal.

**Conclusions:**

The Mix diet appears to be best for minimizing control mosquito mortality, without altering the mosquito survival response following ivermectin ingestion. An unexplained biological phenomenon occurred when ivermectin was ingested in either a blood meal or a plasma meal. The concentration of ivermectin imbibed by the mosquito was lower than that observed in the blood meal, suggesting that some of the ivermectin may be excreted by the mosquito during the blood meal.

**Graphical Abstract:**

**Figure:**
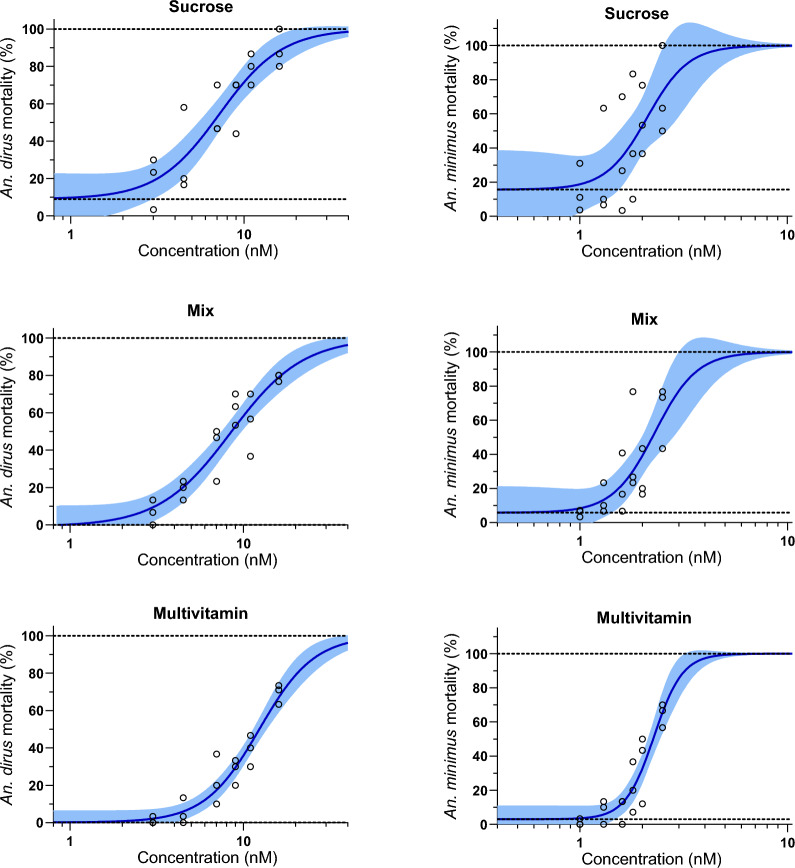

**Supplementary Information:**

The online version contains supplementary material available at 10.1186/s13071-025-07040-2.

## Background

Systemic insecticides are compounds administered to a host that are subsequently ingested by blood-feeding arthropods and can be classified as either endectocides, which have activity against internal and external parasites, or ectocides, which only have activity against external parasites. Systemic insecticides are used widely in veterinary medicine to treat and control a range of external parasites. Several endectocidal and ectocidal compounds have been shown to have mosquito-lethal effects against *Anopheles* mosquitoes, such as ivermectin, eprinomectin, doramectin, fipronil, fluralaner, afoxolaner, and emodepside [1–7]. These endectocidal and ectocidal compounds could be used for vector control by mass administration to livestock to reduce malaria transmission. Ivermectin is an endectocide approved for use in humans, and several laboratory investigations and clinical and veterinary trials have demonstrated the mosquito-lethal effect of ivermectin against *Anopheles* [8–14]. While there have been numerous publications on the subject, there remain some key questions in methodology, which should be addressed to standardize endectocide and ectocidal evaluation across laboratories.

Due to geographical, logistical, and regulatory aspects, it may not be possible to evaluate all *Anopheles* species of interest in the same location where a clinical or veterinary trial is performed. In this case, it would be advantageous to collect blood from the treated host and to transport or ship the blood samples to a secondary laboratory where they can be fed to mosquitoes and survival monitoring performed. However, if a blood sample is frozen, then red blood cells (RBCs) will lyse, and this may not be ideal because RBC lysis is part of the mosquito blood meal digestion process. To avoid RBC lysis, the original blood sample could be centrifuged to separate the plasma from the RBCs, and then the plasma could be frozen and shipped to the secondary laboratory. The plasma could then be thawed and fed directly to mosquitoes. However, since adult mosquitoes use RBCs for their own nourishment [15], and endectocide evaluation entails mosquito survival monitoring, it may be necessary to reconstitute the plasma with freshly collected RBCs.

Laboratories differ in their mosquito-rearing procedures, and one of the differences that can influence mosquito survival is the replacement of sucrose with multivitamin syrup. The rationale for using multivitamin syrup as the sugar source is that it increases male mosquito health, fertility, and overall colony production [16]. Grapefruit juice and other juices high in vitamin C cause inhibition of intestinal CYP3A4, P-glycoprotein, and a range of drug transporters [17]. Ivermectin is metabolized primarily by mammalian cytochrome P450 3A4 [18, 19]. Mosquitoes also possess cytochrome P450 enzymes, which are used to metabolize and detoxify insecticides [20]. Thus, rearing mosquitoes on multivitamin syrup, which contains various vitamins and minerals, could influence the distribution and metabolism of ivermectin and other ingested endectocides, resulting in altered mosquito survival outcomes.

*Anopheles* species have different levels of susceptibility to ivermectin, with *Anopheles dirus* sensu stricto (s.s.) being the most ivermectin-tolerant and *Anopheles minimus* s.s. being the most ivermectin-susceptible, with a four- to sevenfold difference in drug susceptibility [13, 21]. *Anopheles* concentrate RBCs during blood-feeding by excreting plasma, a phenomenon known as pre-diuresis, and it has been shown previously that this rate differs between various *Anopheles* species [22]. Since ivermectin binds avidly to plasma [23], it may be that some of the ivermectin is excreted during pre-diuresis, and it is possible that this may account for differential species tolerance to ivermectin.

Here, we perform several evaluations and experiments with ivermectin to assess the issues discussed above, the influence they may have on mosquito-lethal outcomes, and offer solutions for optimizing methodology for evaluating endectocides and ectocides.

## Methods

### Mosquito rearing

Mosquito colonies were reared at the Insecticide Research Unit in the Department of Medical Entomology, Faculty of Tropical Medicine, Mahidol University in Bangkok, Thailand. *Anopheles dirus* s.s. (Kaw Mai Khaw strain) and *An. minimus* s.s. (Saraburi strain) were reared as described previously [13]. Mosquitoes were reared in the insectary at 28 ± 2 °C, 80 ± 10% relative humidity, and 12 h light:12 h dark photoperiod. Adult colony mosquitoes were provided with 5% multivitamin syrup solution (Seven Seas, PT. Merck Tbk., Jakarta, Indonesia) and 5% sucrose solution (Lin, Thai Roong Ruang Sugar Group Co., Ltd., Petchabun, Thailand) ad libitum.

### Drug dilutions, mosquito blood, and plasma meal preparations

Whole blood was collected from healthy volunteers from two different donor blood types. AB+ blood donors had their plasma separated from RBCs by centrifuging whole blood samples at 3000×*g* for 5 min at 4 °C. Plasma aliquots of 2 ml were stored in cryovials and frozen at −80 °C. Plasma samples from AB+ donors were used to prepare ivermectin dilutions, as AB+ plasma can be mixed with whole blood from any blood donor type without the issue of RBC lysis. Blood donors for mosquito feeds were of any blood type.

Powdered ivermectin compound was obtained from Sigma-Aldrich (St. Louis, MO, USA). Ivermectin was dissolved in dimethylsulfoxide (DMSO) to concentrations of 2 mg/ml, and 12 μl aliquots were frozen at −80 °C. Compounds were thawed, and serial dilutions were made in human AB+ plasma and glass amber vials (Chromacol, Chromacol Ltd., Welwyn Garden City, UK). Final concentrations desired for mosquito membrane-feeding assays were achieved by adding 10 μl of ivermectin-containing plasma dilution to 990 μl of freshly collected blood or plasma. Some ivermectin-spiked blood meals were frozen at −20 °C for later membrane-feeds. Control mosquito meals consisted of previously frozen DMSO diluted in AB+ plasma to match the highest ratio of DMSO fed to mosquitoes in the ivermectin-containing meals.

### Membrane-feeding and survival analysis

Mosquitoes for experiments were 5–7-days post-emergence at the time of membrane-feeding. Approximately 35–40 mosquitoes were gently transferred from emergence cartons via aspiration to clean 0.5 L cylindrical cardboard containers sealed with a mesh screen on top. Mosquitoes were maintained in an upright Accuplus Smart i250 S incubator (Entech Industrial Solution Co, Ltd., Bangkok, Thailand) at 25 ± 1 °C and 80 ± 10% humidity with a 12 h light:12 h dark photoperiod. Mosquitoes were sugar-starved with access to water from 16 to 20 h before their artificial membrane meal.

Glass membrane feeders were placed on top of the mosquito cartons and warmed to 37 °C, and then filled with either plasma or blood (fresh or frozen) ivermectin meals. Mosquitoes were allowed to feed on the membrane for up to 30 min. After the membrane-feeding, up to 30 mosquitoes fed to repletion per container were gently transferred via aspiration to clean cardboard containers (0.5 L). Mosquitoes were maintained in an upright incubator at 25 ± 1 °C and 80 ± 10% humidity with a 12 h light:12 h dark photoperiod. After their blood meal, mosquitoes were provided 10% sucrose (Lin, Thai Roong Ruang Sugar Group Co., Ltd., Petchabun, Thailand) ad libitum. Mosquito survival was monitored daily for 10 days, and any dead mosquitoes were removed by aspiration and recorded. Ten days after the blood meal, any live mosquitoes were recorded as alive and then frozen.

### Experimental design

The first experiment evaluated the impact of sugar-feeding regimen prior to blood meal ingestion on mosquito survival. Groups of pupae from the same colony cohorts were placed into separate emergence cages, and adults were provided three different sugar diets prior to experimental membrane-feeding, as follows: (1) “Sucrose,” 10% sucrose until used in experiments; (2) “Mix,” 2 days of 5% multivitamin syrup with 5% sucrose, followed by 10% sucrose until used in experiments; and (3) “Multivitamin,” 5% multivitamin syrup with 5% sucrose until used in experiments. Cotton balls were soaked in the sugar diet solution and placed on top of the mesh screen of the adult cages, and the sugar solution was replaced daily. Blood meals across a range of ivermectin concentrations were blood-fed to *An. dirus* (0, 3.0, 4.5, 7.0, 9.0, 11.0, 16.0 nM) and *An. minimus* (0, 1.0, 1.3, 1.6, 1.8, 2.0, 2.5 nM). Following their blood meal, mosquitoes were provided 10% sucrose ad libitum.

The second experiment evaluated the impact of ingesting ivermectin-spiked fresh plasma, fresh blood, or frozen blood on mosquito survival. Ivermectin-containing meals across a range of ivermectin concentrations were fed to *An. dirus* (0, 4.5, 7.0, 9.0, 11.0 nM) and *An. minimus* (0, 1.3, 1.6, 2.0 nM). Some ivermectin-spiked blood meals were frozen at −20 °C for later membrane-feeds and classified as frozen blood. Freshly prepared plasma-spiked and blood-spiked meals, and previously frozen blood-spiked meals, were fed to separate groups of mosquitoes from the same cohort. All mosquitoes for these experiments were raised on the Mix sugar diet. The number of successfully blood-fed mosquitoes was also recorded from each container to assess the feeding rate on fresh plasma, fresh blood, or frozen blood. After their blood meal, mosquitoes were provided 10% sucrose ad libitum.

The third experiment aimed to quantify ivermectin concentrations from mosquito blood meals and midguts of the mosquitoes. Firstly, the average blood meal volume during blood-feeding was determined for each species. Sixty unfed and 60 blood-fed female *An. dirus* or *An. minimus* mosquitoes were placed individually into microcentrifuge tubes and frozen at −20 °C for 1 h. Before measurement, the mosquitoes were thawed at ambient temperature. Each tube was weighed using a microbalance (Sartorius, MC5), and the weight of the empty tube was subtracted. To estimate the blood meal volume inside the mosquito midgut, the difference in average weight between the 60 unfed and 60 blood-fed mosquitoes was divided by 1.05 µg, the specific gravity of blood [24], to obtain the gravimetric blood volume in microliters. Secondly, the concentration of ivermectin imbibed by blood-feeding mosquitoes was assessed. Fresh blood meals at varying concentrations of ivermectin (5 ng/ml [4.37 nM], 50 ng/ml [43.72 nM], 200 ng/ml [174.86 nM]) were provided to groups of 40 *An. dirus* or 40 *An. minimus* mosquitoes. The membrane-feeds were terminated once most of the mosquitoes were fully engorged, usually around 10–15 min after starting the feed, and before mosquito diuresis (i.e., post-feed excretion) occurred. A 120 µl aliquot of each mosquito blood meal was frozen at −80 °C for subsequent liquid chromatography–tandem mass spectrometry (LC–MS/MS) analysis. Mosquitoes were subjected to a quick knockdown in a −20 °C freezer for 5 min. Following knockdown, mosquitoes were transferred to glass Petri dishes lined with clean filter paper, kept on wet ice, and stored in a refrigerator at 4 °C . Batches of mosquitoes on ice were placed under a stereomicroscope (Carl Zeiss, Stemi 2000-C). A drop of 1× phosphate-buffered saline (PBS) was applied to a glass slide. During dissection, a single mosquito was carefully placed onto the slide, and the legs and wings were removed with forceps. An incision was made with minuten pins at the second- or third-to-last abdominal segment. The segment was then grasped with forceps and gently pulled from the abdomen. The midgut was gently pulled from the abdomen without rupturing the midgut, and the anterior midgut was detached by snipping with the forceps. Ten mosquito midguts were dissected and pooled into a centrifuge tube, and samples were frozen at −80 °C for later LC–MS/MS analysis.

### Extraction procedure

Blood (20 µl) from mosquito blood meals (three replicates for each ivermectin concentration: 4.37, 43.72, 174.86 nM) and blood calibrators (duplicates for each ivermectin concentration: 0.84, 2.96, 10.40, 36.37, 127.65, and 335.73 nM) were aliquoted into microcentrifuge tubes. Acetonitrile (90:10, v/v) (100 µl) with stable isotope-labeled internal standard ivermectin-D2 (61.20 nM) was added to each tube. Samples were vortexed for 1 min, then centrifuged at 10,000 rpm (9391×*g*) for 5 min to separate the supernatant from precipitated RBCs. An 80 µl aliquot of the supernatant was transferred to a 100 µl Polyspring glass vial insert.

Dissected midguts were thawed, and midgut blood was aspirated with a pipette from the pooled group of 10 midguts, with approximately 7 µl from *An. minimus* and 20 µl from *An. dirus*. As the midgut blood is much more viscous than normal blood, likely due to RBC concentration during mosquito blood-feeding, only a single sample extraction was performed. To aid pipetting, 10 µl of water was added to the tube. Acetonitrile (90:10, v/v) (90 µl) with stable isotope-labeled internal standard ivermectin-D2 (61.20 nM) was added to each tube. Samples were vortexed for 1 min, then centrifuged at 10,000 rpm (9391×*g*) for 5 min to separate the supernatant from precipitated RBCs. An 80 µl aliquot of the supernatant was transferred to a 100 µl Polyspring glass vial insert.

### Liquid chromatography–tandem mass spectrometry (LC–MS/MS)

The LC system was a Dionex UltiMate 3000 system consisting of an LC pump with a vacuum degasser (LPG-3400RS), a temperature-controlled micro-well plate autosampler set at 10 °C (WSP-3000TBRS), and a temperature-controlled column compartment set at 40 °C (TCC-3000RS) (Thermo Scientific, MA, USA). Data acquisition and processing were performed with an Analyst 1.7 (Sciex, MA, USA). The analytes were separated on an InfinityLab Poroshell 120 EC-C18 column (50 mm × 3.0 mm, I.D. 2.7 µm) (Agilent Technologies, CA, USA, 699975-302), with a pre-column C18 AJO-7596 (4 mm × 2 mm, I.D. 3.0 µm) (Phenomenex, Torrance, CA, USA) at a flow rate of 500 µl/min under isocratic conditions using 100% mobile phase A (acetonitrile/ammonium formate 2 mM with 0.5% formic acid, 90:10, v/v) for 2.10 min (0–2.10 min). This was followed by a washout with 100% mobile phase B (methanol/acetonitrile, 75:25, v/v) for 1 min (2.10–3.10 min), after which the mobile phase was immediately returned to 100% A and allowed to equilibrate (3.10–5 min) until the next sample injection. The total runtime was 5 min per sample, with an injection volume of 5 µl.

An API 5000 triple quadrupole mass spectrometer (Sciex, Framingham, MA, USA) with a Turbo V ionization source interface in positive ion mode was used for the MS/MS analysis. The ion spray voltage was set to 5500 V, and the drying temperature was 450 °C. The curtain, nebulizer, and auxiliary gases were 30, 45, and 50 psi, respectively. All collision energy was set to 35 V. Quantification was performed using selected reaction monitoring for the specific transitions of ivermectin (m/z 892.5 → 307.1) and ivermectin-D2 (m/z 894.5 → 309.1).

### Statistical analyses

The lethal concentrations killing 50% (LC_50_) and 90% (LC_90_) of mosquitoes from sugar-feeding experiments were estimated using a normalized concentration–response analysis (half maximal inhibitory concentration [IC_50_]) with a variable slope (Hill), assuming a maximum of 100% mosquito mortality and an estimated baseline mosquito mortality (i.e., mosquito mortality at zero drug concentration). Mosquito survival curves for each species and membrane meal type (fresh plasma, fresh blood, or frozen blood) at each ivermectin concentration were compared using log-rank survival curve analysis (Mantel–Cox method). Significant differences for log-rank survival curve analysis were set at *P* < 0.05. All mosquito survival analyses were performed using GraphPad Prism v.9.5.0 (GraphPad Software, Inc., San Diego, CA, USA).

## Results

### Impact of pre-blood meal sugar regimen on mosquito mortality

**Fig. 1:**
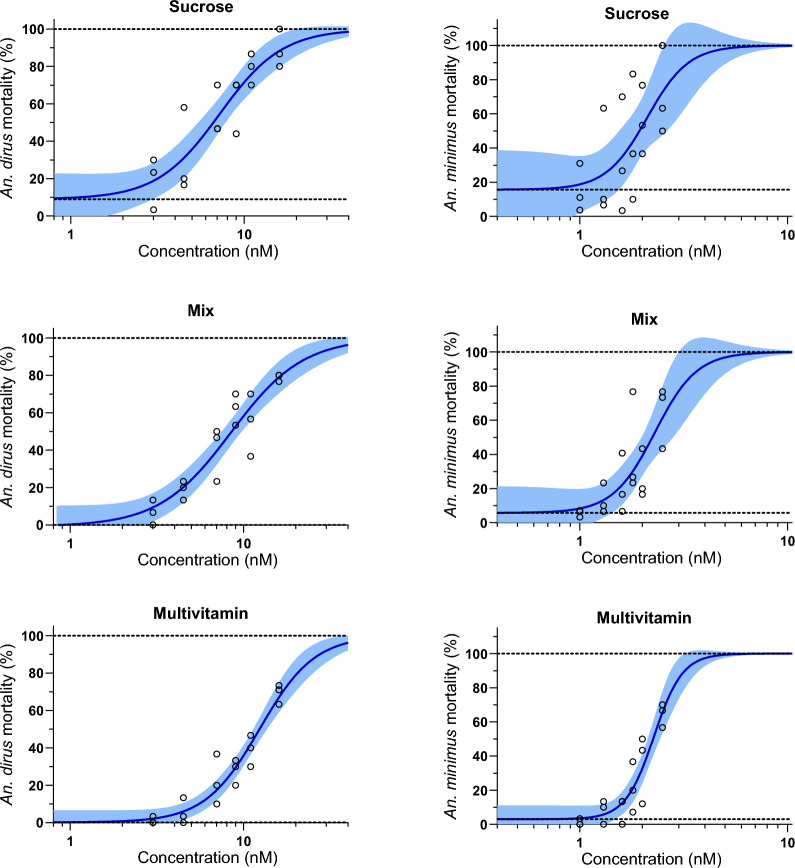
*Anopheles dirus* (left panels) and *Anopheles. minimus* (right panels) cumulative mortality when raised on Sucrose (upper panels), Mix (middle panels), and Multivitamin (lower panels) sugar regimens prior to an ivermectin blood meal. Circles represent cumulative mosquito mortality at 10 days after blood meal ingestion. Solid blue lines represent the mean concentration–response relationship, and the shaded area represents the 95% confidence interval associated with the nonlinear fit. Dashed black lines represent the fixed maximum effects of 100% mortality and the estimated minimum effect associated with baseline mortality observed from control mosquitoes.

Three replicates with a total of 1888 *An. dirus* mosquitoes were used to calculate the LC_50_ and LC_90_ values for ivermectin when raised on different sugar diets: Sucrose (*n* = 627), Mix (*n* = 630), Multivitamin (*n* = 631). Three replicates with a total of 1825 *An. minimus* were used to calculate the LC_50_ and LC_90_ values for ivermectin when raised on different sugar diets: Sucrose (*n* = 618), Mix (*n* = 623), Multivitamin (*n* = 584). For *An. dirus*, the LC_50_ values were equivalent for Sucrose and Mix, but substantially higher for Multivitamin (Table 1). For *An. minimus*, the LC_50_ values were equivalent for Sucrose, Mix, and Multivitamin (Table 1). For both *An. dirus* and *An. minimus*, the control mortality was highest for the Sucrose diet compared to Mix and Multivitamin diets (Fig. 1).

**Table 1:** *Anopheles dirus* and *Anopheles minimus* ivermectin susceptibility when raised on Sucrose, Mix, and Multivitamin diets prior to an ivermectin-containing meal

Species	Sugar regimen	Baseline mortality [95% CI] (%)	LC_50_ [95% CI] (nM)	LC_90_ [95% CI] (nM)	Hill slope
*An. dirus*	Sucrose	9.01 [−5.49 to 22.23]	7.22 [5.67–8.85]	18.00 [13.21–28.44]	2.40
	Mix	−0.85 [−12.01 to 9.49]	8.48 [7.10–10.06]	24.17 [18.06–36.43]	2.10
	Multivitamin	0.16 [−6.62 to 6.23]	12.38 [11.26–13.67]	27.57 [22.12–37.62]	2.74
*An. minimus*	Sucrose	15.64 [−10.10 to 35.03]	2.11 [1.64–3.35]	3.49 [2.34–16.64]	4.37
	Mix	5.77 [−11.58 to 19.75]	2.30 [1.98–2.93]	3.83 [2.84–8.56]	4.30
	Multivitamin	3.02 [−5.12 to 10.32]	2.28 [2.13–2.46]	3.22 [2.82–4.03]	6.30

*CI* confidence interval, *nM* nanomolar, *LC*_*50*_ lethal concentration killing 50% of mosquitoes, *LC*_*90*_ lethal concentration killing 90% of mosquitoes

### Impact of membrane meal type on mosquito feeding propensity

**Fig. 2:**
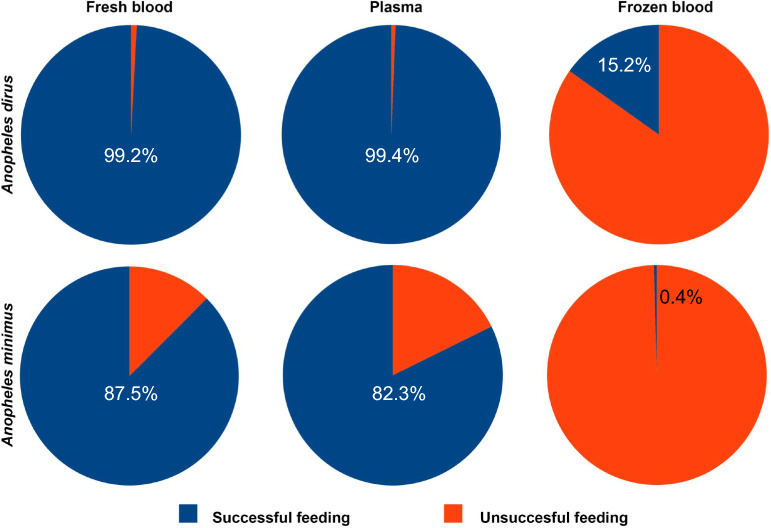
Proportion of *Anopheles dirus* (top row) and *An. minimus* (bottom row) blood-feeding success when offered membrane meals with fresh blood, fresh plasma, or previously frozen blood. Blue indicates the proportion of successful blood-feeding, while red indicates the proportion of unfed mosquitoes. Numbers indicate the percent of mosquitoes which successfully obtained a blood meal

Three replicates with a total of 1440 *An. dirus* mosquitoes were used to evaluate the mosquito feeding propensity on fresh plasma (*n* = 480), fresh blood (*n* = 480), and frozen blood (*n* = 480). Three replicates with a total of 1440 *An. minimus* mosquitoes were used to evaluate the mosquito feeding propensity on fresh plasma (*n* = 480), fresh blood (*n* = 480), and frozen blood (*n* = 480). For both *An. dirus* and *An. minimus*, there was a substantial reduction in the likelihood of feeding when offered previously frozen blood (Fig. 2), with nearly no *An. minimus* feeding on this blood source. There was no substantial difference in the probability of feeding on plasma or whole blood within the two species evaluated, but there was a reduced feeding success for *An. minimus* in general compared to *An. dirus* (Fig. 2).

### Impact of membrane meal type on mosquito mortality

**Fig. 3:**
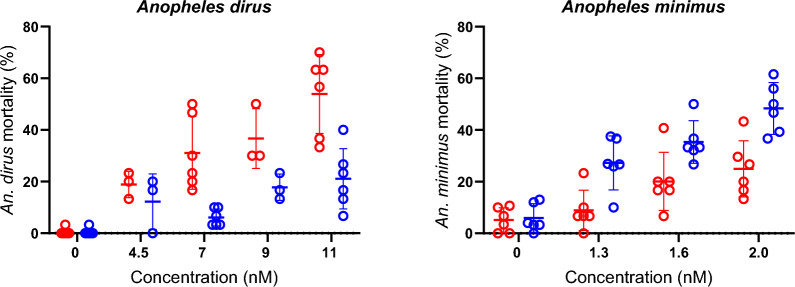
Cumulative percent mortality of *Anopheles dirus* (left panel) and *Anopheles minimus* (right panel) at day 10. Mosquitoes were membrane-fed ivermectin-spiked fresh blood meal (red circles) or a fresh plasma meal (blue circles). Different ivermectin concentrations (nM) were fed to mosquitoes as indicated on the *x*-axis. Lines depict the mean and standard deviation

Six replicates with a total of 1430 *An. dirus* mosquitoes were used to evaluate the mortality difference between ivermectin-spiked blood or plasma meals across four different ivermectin concentrations (4.5, 7.0, 9.0, 11.0 nM). Note that because of a dilution calculation error, the 4.5 nM and 9 nM concentrations only had three replicates performed. Six replicates with a total of 1386 *An. minimus* mosquitoes were used to evaluate the mortality difference between ivermectin-spiked blood or plasma meals across three different ivermectin concentrations (1.3, 1.6, 2.0 nM). Ivermectin was significantly more lethal to *An. dirus* when ingested in a blood meal than in a plasma meal at 7.0, 9.0, and 11.0 nM, but not the 4.5 nM concentration, which was not significantly different. The opposite occurred for *An. minimus*, as ivermectin was significantly more lethal in a plasma meal than in a blood meal at all concentrations evaluated. Control blood meals were not significantly different between plasma and blood meals for either species (Table 2; Fig. 3; Additional file 1: Fig. S1).

**Table 2:** *Anopheles dirus* and *Anopheles minimus* survival curve (Mantel–Cox) comparison between mosquitoes that fed on ivermectin-spiked plasma meals or fresh blood meals

Species	Ivermectin	*χ*^2^	*P*-value	Reps.	*N*
*An. dirus*	Control	0.00	0.9693	6	350
	4.5 nM	1.45	0.2286	3	180
	7 nM	38.47	< 0.0001*	6	360
	9 nM	9.79	0.0018*	3	180
	11 nM	50.03	< 0.0001*	6	360
*An. minimus*	Control	0.02	0.8862	6	338
	1.3 nM	18.83	< 0.0001*	6	347
	1.6 nM	9.39	0.0022*	6	355
	2.0 nM	19.94	< 0.0001*	6	346

*χ*^2^ Chi-square, *Reps*. replicates, *N* number of mosquitoes, *nM* nanomolar. * Significant at *P* < 0.05

### Ivermectin concentrations in blood meals and mosquito midguts

The average blood meal volume was 3.78 µl for *An. dirus* and 1.27 µl for *An. minimus*. Based on these measurements, it was estimated that approximately eight blood-fed *An. dirus* midguts and 20 blood-fed *An. minimus* midguts needed to be harvested to collect sufficient midgut blood volume to meet the minimum volume needed for extraction (i.e., 20 µl). However, due to the high viscosity of midgut blood compared to normal blood, likely caused by RBC concentration during blood-feeding, it was decided that water (10 µl) should be added to make pipette manipulation more feasible. Furthermore, the midgut dissection process was time-consuming; thus, it was decided that only 10 mosquito midguts should be harvested from each blood meal.

**Fig. 4:**
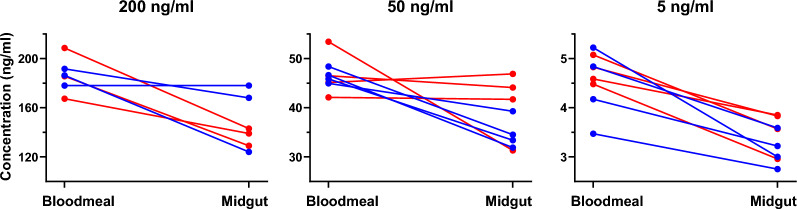
The difference in ivermectin concentrations from blood meals and midguts. Circles represent the measured concentration of ivermectin (ng/ml) in the blood meal (left) and the midgut (right). Blue circles are concentrations associated with *An. minimus*, and red circles are concentrations associated with *An. dirus*. The connecting lines link the concentrations found in the original blood meal to the concentrations found in the midguts of mosquitoes that fed on the same blood meal

A total of 11 blood-feeds were evaluated for each species: three at 200 ng/ml, four at 50 ng/ml, and four at 5 ng/ml. For *An. dirus*, a 20.3% ± 4.4% reduction in ivermectin concentration was measured from the blood meal compared to the midgut, and for *An. minimus*, a 23.4% ± 3.5% reduction was found (Fig. 4), indicating no difference in ivermectin concentration ingested into the midgut between the two species. A total of 22 blood meal samples were analyzed in triplicate by LC–MS/MS. The difference between target blood meal concentrations and the LC–MS/MS-measured ivermectin concentrations revealed that the latter was 7.3% ± 1.8% lower.

## Discussion

The work presented here attempts to optimize methodology for endectocide and ectocide evaluation by assessing different factors which may influence experimental and mosquito-lethal outcomes.

In Thailand, it is very common to supplement mosquito colony sugar sources with multivitamin syrup [16]. While this may be ideal to maximize colony health and reproductive output, it was observed anecdotally that there was a differential impact on *An. minimus* survival if multivitamin or 10% sucrose solution was provided during ivermectin evaluations [25]. Thus, it is important to evaluate this potential interaction further. For *An. dirus* provided with a Multivitamin diet prior to ivermectin, there was a substantial increase in the ivermectin LC_50_ compared to Sucrose and Mix diets, but this was not observed with *An. minimus* (Table 1). It should be noted that there was more variability in the observed mortality data and that the Hill slope was higher for *An. minimus* compared to *An. dirus,* which indicates a steeper “on/off” mortality effect of ivermectin for *An. minimus*, and therefore more difficult to characterize potential differences among sugar regimens for *An. minimus*. For both *An. dirus* and *An. minimus*, the control mosquito mortality was highest for the Sucrose diet compared to Mix and Multivitamin diets. In endectocide and ectocide evaluations, it is important to minimize control mosquito mortality to reduce variation in mortality analysis outcomes. Thus, based on this experimental design, we recommend that mosquitoes be raised on the Mix sugar diet for 48 h post-emergence and then 10% sucrose thereafter. While the Seven Seas Brand multivitamin syrup used here does not contain vitamin C, there is an impact on mosquito survival when the Multivitamin diet is ingested before ivermectin-containing blood meals, suggesting that other vitamins and minerals may affect mosquito-lethal outcomes for ivermectin. It should be noted that other multivitamin syrups may cause different impacts on survival and mosquito-lethal outcomes. These findings here may be important to explore further, particularly how wild sugar sources (e.g., extrafloral nectaries) may influence mosquito survival after ingestion of ivermectin or other endectocides and ectocides.

As insectaries may not always be on-site in human or veterinary trials with endectocides or ectocides, samples may need to be sent off-site to evaluate mosquito-lethal effects. Since RBCs will lyse when frozen, and RBC lysis is part of the mosquito blood-feeding and digestion process, this could potentially impact mosquito survival or mosquito-lethal outcomes during endectocide or ectocide evaluation. Thus, we wanted to compare whether fresh plasma, fresh blood, or previously frozen blood spiked with ivermectin altered mosquito-lethal outcomes. Surprisingly, previously frozen blood dramatically reduced the mosquito feeding rate to less than 20% for *An. dirus* and inhibited nearly all *An. minimus* blood-feeding (Fig. 2). Thus, further evaluation of frozen blood on the mosquito-lethal effect could not be performed. Based on this, we recommend that human or veterinary trials shipping samples off-site for mosquito evaluation elsewhere should collect plasma at the trial site, then reconstitute with fresh RBCs at the mosquito evaluation site. An important factor to consider with this arrangement is that mixing certain human blood group types will cause lysis of RBCs. This is a well-established concern for human blood, platelet, or plasma transfusion. In humans, an O-donor for RBCs will allow for universal mixing with plasma from any blood group (A, B, AB, O) and type (Rh+ , Rh−) [26]. However, in some human populations (e.g., Asian), the Rh type and especially the O blood group can be exceedingly rare, less than 0.1% in Thai populations [27], making it difficult to find O blood donors. When performing endectocide and ectocide dilutions in human plasma, the plasma donor AB+ group can be used, as its addition to RBCs with any blood group or blood type will not cause RBC lysis [26]. In cattle, there are over 500 different blood groups, but hemolysis has not been observed when mixing across blood groups [28, 29], obviating this concern. Mixing plasma and RBCs from different species may require further evaluation to determine the impact on RBC lysis and mosquito-lethal outcomes. Another issue to take into consideration with this approach is the dilution of the original sample by adding RBCs, which must be taken into consideration in the data analysis of mosquito mortality data.

Interestingly, there was a dramatic reversal in mortality response, with ivermectin-spiked blood being substantially more lethal to *An. dirus*, whereas ivermectin-spiked plasma was substantially more lethal to *An. minimus* (Table 2; Fig. 3; Additional file 1: Fig. S1). This demonstrates that it is critical to reconstitute ivermectin-containing plasma with freshly collected RBCs to achieve similar mosquito-lethal outcomes as the original whole blood may have induced. This RBC reconstitution of plasma should be done at the approximate hematocrit of the target host, which is 45% for humans and 35% for cattle [29]. One possible explanation for this biological difference in plasma- and blood-induced mortality could be due to species-specific differences in pre-diuresis during feeding. Pre-diuresis is the process of anal excretion of liquids, mostly plasma, during blood-feeding, which is done to concentrate the RBCs and only occurs in *Anopheles* mosquitoes. The rate of RBC concentration differs among *Anopheles* species, and not all mosquitoes feeding on the same blood source may perform pre-diuresis. It was further hypothesized that this rate of pre-diuresis and RBC concentration may be driven by the host feeding preference of the *Anopheles* species, as different host species have varied RBC sizes (humans, 87 µm^3^; cattle, 59 µm^3^) [22]. These potential *Anopheles* species differences in pre-diuresis could lead to different amounts of ivermectin being imbibed, as ivermectin is highly bound to human plasma with 93% affinity [23]. Although not directly measured, it did not appear that there was a diversion of the plasma meal from the midgut to the crop, as mosquito abdomens of plasma-fed mosquitoes were distended in a similar manner as blood-fed mosquitoes.

For both *An. dirus* and *An. minimus,*, there was an approximately 20% loss of ivermectin concentration between the blood meal and midgut (Fig. 4). Thus, the approximately fourfold difference in ivermectin susceptibility between *An. dirus* and *An. minimus* is likely not driven by differing amounts of ivermectin ingestion between the two species. The blood-feeds were terminated before the mosquitoes could diurese (i.e., defecate), suggesting that this loss of ivermectin was due to excretion of ivermectin in fluids during pre-diuresis. To further support this, in *Aedes aegypti*, a mosquito species that does not pre-diurese, there was a 1:1 ratio of ivermectin in the blood meal and whole individual mosquitoes after blood-feeding [30]. The equipment used in the *Ae. aegypti* evaluations was more sensitive, allowing for quantification of ivermectin from individual mosquitoes, while the *Anopheles* evaluation here had to use batches of 10 mosquito midguts to quantify ivermectin. Since not all mosquitoes may pre-diurese, even when feeding on the same host source [22], this could introduce further variation in the amount of ivermectin imbibed by an individual *Anopheles* mosquito. However, based on the 1:1 ratio observed in *Ae. aegypti* [30], it is unlikely that an *Anopheles* mosquito would end up ingesting a higher concentration of ivermectin than the original blood meal source. In both the *Anopheles* and *Ae. aegypti* evaluations, the mosquitoes were fed via membrane-feeding; it would be of interest to assess differences between blood meal and midgut concentrations from several treated hosts and with direct mosquito feeding.

A final point to note on methodology is that preparing ivermectin dilution series in human plasma and amber glass vials produced a reasonable level of accuracy for the final blood meal target concentrations, differing only by a 7.3 ± 1.8% reduction. This is important, as previous experiments with ivermectin dilutions in PBS and plastic centrifuge tubes led to final blood meal concentrations reduced by 20–80% from the intended target concentration [12]. The reduction in ivermectin concentration with PBS dilutions was likely caused by non-specific plastic binding. Since ivermectin binds avidly to human plasma proteins [23], diluting ivermectin in plasma reduces the potential for non-specific plastic binding in the pipette tip or dilution tube, and using glass amber vials further reduces this possibility.

A limitation of this study is that ivermectin was the only compound evaluated. However, since ivermectin is the most commonly used endectocide, is approved for human use, and is the most frequently evaluated endectocide for *Anopheles* mosquito-lethal effect in laboratory settings, it is the most critical endectocide to evaluate. It is likely that the findings here with ivermectin could be extrapolated to other endectocides or ectocides, but this would require further evaluation to confirm.

## Conclusions

These laboratory experiments were designed to optimize in vitro methods for endectocide and ectocide evaluations to assess *Anopheles* mosquito-lethal effects. The Mix sugar diet (i.e., multivitamin syrup plus 5% sucrose) for 48 h post-emergence, followed by 10% sucrose for the remainder of the experiment, appears ideal to minimize control mosquito mortality while not altering ivermectin-induced mortality outcomes. When evaluating the impact of endectocide or ectocide treatment in a facility separate from where the human or animal trial was performed, plasma should be frozen at the trial site and later reconstituted with RBCs at the mosquito evaluation site. When preparing endectocide and ectocide dilution series, it is strongly recommended to dilute in plasma and glass vials. *Anopheles* mosquitoes appear to retain less ivermectin after the blood meal than the host blood source by approximately 20%, but there was no difference found between *An. dirus* and *An. minimus*.

## Supplementary Information

Additional file 1.

## Data Availability

All data supporting the main conclusions of this study are included in the manuscript and supplementary file.

## Abbreviations

DMSO: Dimethylsulfoxide
PBS: Phosphate-buffered saline
RBC: Red blood cell
LC_50_: Lethal concentration that kills 50% of mosquitoes
LC_90_: Lethal concentration that kills 90% of mosquitoes
LC–MS/MS: Liquid chromatography–tandem mass spectrometry
