# Supplementary material for: Optimizing endectocide and ectocide compound evaluation in Anopheles malaria vectors
Source: Parasit Vectors. 2025 Oct 17;18:417. doi: 10.1186/s13071-025-07040-2 (PMC12534914; doi:10.1186/s13071-025-07040-2)
Supplement: Supplementary file 1 — Additional file 1. [file 13071_2025_7040_MOESM1_ESM.docx]

**Supplemental Information**


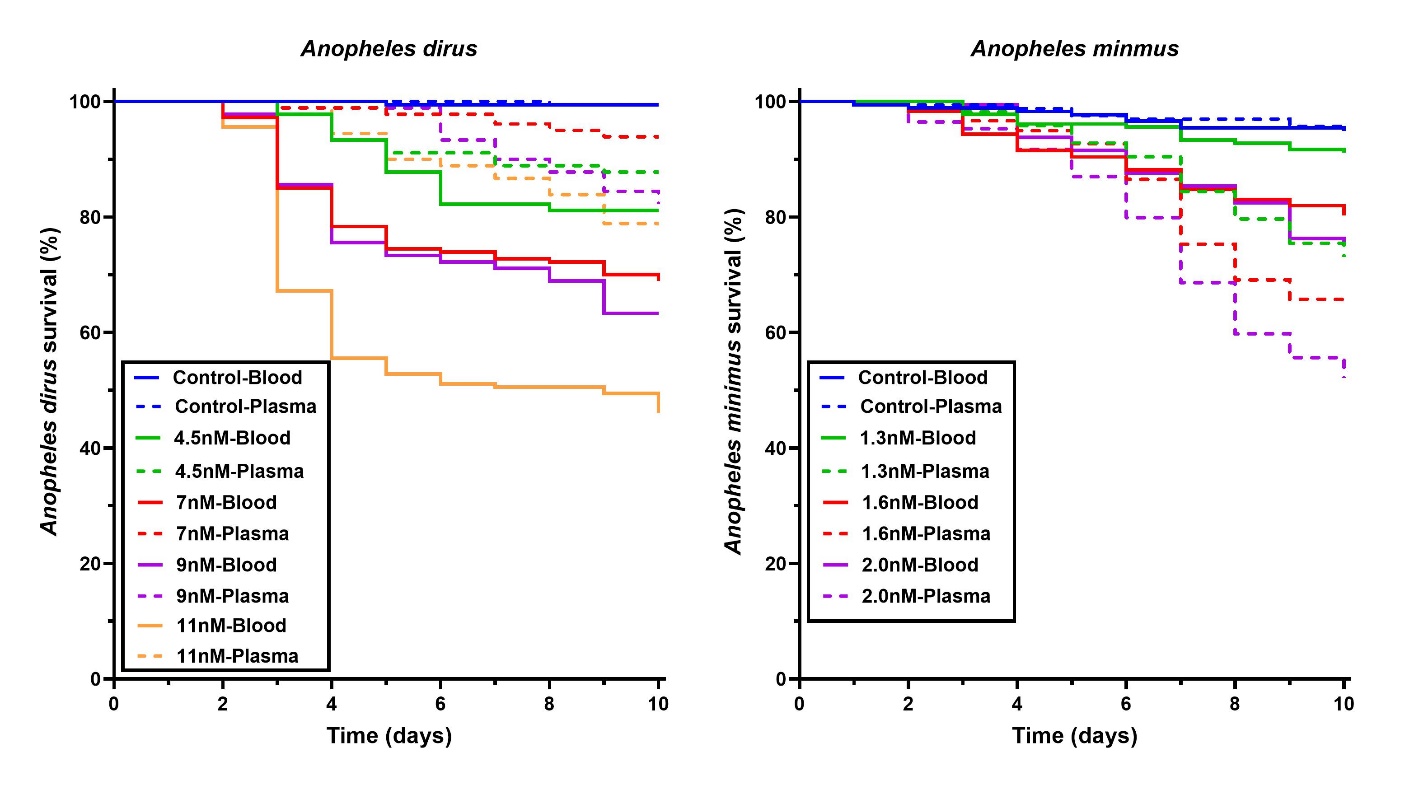


**Fig. S1.** Percent survival of *Anopheles dirus* (left panel) and *Anopheles minimus* (right panel) are depicted relative to time of ivermectin ingestion when membrane fed a fresh blood meal (solid lines) or a plasma meal (dashed lines) containing ivermectin. Different ivermectin concentrations (nM) were fed to mosquitoes as indicated in the legend for each species.
